# Interventions to decrease skin cancer risk in outdoor workers: update to a 2007 systematic review

**DOI:** 10.1186/1756-0500-7-10

**Published:** 2014-01-07

**Authors:** Caitlin Horsham, Josephine Auster, Marguerite C Sendall, Melissa Stoneham, Philippa Youl, Phil Crane, Thomas Tenkate, Monika Janda, Michael Kimlin

**Affiliations:** 1 School of Public Health and Social Work, Institute of Health and Biomedical Innovation, Queensland University of Technology, Victoria Park Road, Kelvin Grove 4059, Queensland, Australia; 2 Curtin University, Perth, Australia; 3 Cancer Council Queensland, Brisbane, Queensland, Australia; 4 School of Occupational and Public Health, Ryerson University, Toronto, Canada

**Keywords:** Skin neoplasms, Melanoma, Outdoor workers, Occupational exposure, Primary prevention

## Abstract

**Background:**

Outdoor workers are at high risk of harmful ultraviolet radiation exposure and are identified as an at risk group for the development of skin cancer. This systematic evidence based review provides an update to a previous review published in 2007 about interventions for the prevention of skin cancer in outdoor workers.

**Results:**

This review includes interventions published between 2007-2012 and presents findings about sun protection behaviours and/or objective measures of skin cancer risk. Six papers met inclusion criteria and were included in the review. Large studies with extended follow-up times demonstrated the efficacy of educational and multi-component interventions to increase sun protection, with some higher use of personal protective equipment such as sunscreen. However, there is less evidence for the effectiveness of policy or specific intervention components.

**Conclusions:**

Further research aimed at improving overall attitudes towards sun protection in outdoor workers is needed to provide an overarching framework.

## Background

Skin cancer is one of the most common cancers diagnosed worldwide, especially in fair-skinned populations, and incidence and mortality have increased over the past decade [1]. The most invasive form of skin cancer, melanoma, has a high mortality rate, particularly when not detected early [1]. Non-melanoma skin cancers (NMSCs), such as basal cell carcinoma (BCC) and squamous cell carcinoma (SCC) are more common but less likely to metastasize, with only a small proportion leading to mortality [1]. However, NMSCs lead to high patient morbidity and financial burden on healthcare systems in Australia, for example, over 700,000 NMSCs were treated in 2010, resulting in a cost to the health system of around $511 million [2].

The most important contributing factor to skin cancer development is exposure to ultraviolet radiation (UVR) coupled with a fair skin type [1,3,4]. Outdoor workers are at risk of UVR exposure due to the nature of their occupation, high levels of UVR exposure, and have been identified as an at risk group for the development of skin cancer [5]. While the term ‘outdoor worker’ has been defined in varying ways in the literature, broadly speaking it includes workers who work outdoors for 3 or more hours on a usual workday. This may include workers in industries such as building and construction, transport, or agriculture. Several recent studies have used dosimetry methods to objectively measure UVR exposure in workers, finding that outdoor workers receive higher than recommended doses [6-9], and significantly larger doses compared to indoor workers [10]. Other studies have identified that outdoor workers are likely to spend significant amounts of time in the sun during non-work hours [11,12]. Two meta-analyses demonstrated a clear association between outdoor work and increased risk of SCC [13] and BCC [14]. There may be a link between risk of developing melanoma and outdoor work although the evidence is less clear (reviewed in [15]).

Exposure to UVR can be reduced by preventative strategies, however, a body of cross-sectional evidence suggests the uptake of strategies among outdoor workers is low [9,16,17]. Recommended strategies include using protective measures such as wide-brimmed hats, long-sleeved shirts and pants, sunglasses and sunscreen; and avoiding the sun by seeking shade and rescheduling work tasks outside of peak UV times (10 am – 3 pm). An Australian study of construction workers found just 10% were using adequate sun protective equipment [9]; and a number of other studies have found the use of similar protective equipment to be low among outdoor workers (reviewed in [16]). For example, a recent study of British construction workers found only 23% wore wide-brimmed hats while at work, and 51% wore long-sleeved shirts, although more workers wore sunscreen (60%) [17]. In a sample of US transport workers just 19% reported regular use of sunscreen, and 17% reported regularly wearing long-sleeved shirts [18]. In regards to seeking shade and rescheduling work tasks, both have also been reported to be infrequently used by outdoor workers to reduce UVR exposure [9,17,19,20].

There is a need to improve sun safe behaviour amongst outdoor workers, and organisations employing such workers have both a responsibility and opportunity to promote this behaviour. Diepgen et al. [5] argue there is enough evidence to consider skin cancer an ‘occupational disease’. In Australia, while UVR is not explicitly mentioned in national workplace health and safety legislation, employers can be held liable for skin cancer developed as a result of occupational exposure, unless they ensure adequate precautions are taken to prevent the risk [21]. Workplace policy may determine when and what type of preventative measures can be taken [16]. Furthermore, organisations have the power to make environmental changes which support preventative behaviour, such as providing shade structures. Finally, the workplace provides an ideal context in which sun protection can be promoted and modelled, through education, supervision and awareness raising activities [22].

Organisational and community-based intervention studies to decrease skin cancer risk among outdoor workers have previously been systematically reviewed [16]. The review included eight intervention studies conducted between 1966 and 2007, most involving a suite of intervention components [16]. Only one study mentioned an organisational sun protection policy component [23]. Three included an environmental and/or structural component, such as providing shade structures [23-25]; and four also included the provision of some kind of PPE (personal protective equipment), such as sunscreen and/or sunglasses and brimmed hat [23,24,26,27]. Two involved a skin screening component [28,29]. All studies included an educational and/or awareness raising component, with one example being how to use shade to reduce sun exposure [23-30]. Some of the interventions reviewed, particularly those with multiple components, produced promising results. However, Glanz et al. [16] concluded that due to study limitations such as too few well designed studies, there was insufficient evidence to demonstrate the effectiveness of any intervention in promoting sun safe behaviour or decreasing UV exposure in this population.

The purpose of this paper is to provide further evidence for interventions to reduce sun exposure and/or its harmful effects in outdoor workers; via a systematic review of relevant studies published between 2007 and 2012.

## Methods

Search terms and inclusion criteria were adapted from Saraiya et al. [31]. A search of the online databases MEDLINE, Cumulative Index to Nursing and Allied Health Literature (CINAHL), and PsycInfo was made using terms related to skin neoplasms, UVR exposure, intervention, and outdoor workers. Reference lists of relevant papers were manually searched for further studies. Papers were required to meet the following criteria: published between July 2007 and December 2012; evaluate an intervention for prevention of skin cancer in outdoor workers; written in English; be a primary study rather than; for example; a guideline or review; provide outcomes on sun protection behaviours and/or objective measures of skin cancer risk (example outcomes include: increases in worker knowledge, attitudes and intentions to reduce UV exposure; reduction of sunburn; behavioural changes in sun protection habits and changes in workplace policies and environments to reduce exposure); and compare a group of people exposed to the intervention with a group who had not been exposed or had been less exposed (could be concurrent comparison or comparison of same group over time). Papers were screened by two independent reviewers for eligibility for inclusion. Data were extracted in several categories as shown in Table 1.

**Table 1:** Extracted data from intervention studies included in the review

**Author, date, design, duration**	**Population & sample size**	**Intervention**	**Results summary**	**Limitations**	**Evidence level**^**a**^
Stock et al., 2009 [18]	n = 148 (97.3% retention at 12 months)	2-component intervention; photo taken with UV filter camera, and educational video on sun protection and either skin cancer or photoageing	Significantly great increase in sun protection score *(combined self-reported use of sunscreen, hat, long-sleeves; and objective skin tanning measure)* at 12 months in groups #3-5 (+9%; +21%; +14% respectively) compared to control (−17%) & group #2 (−11%)	Small sample size per group; limited variation in gender/ethnicity	II
Randomised controlled trial (RCT)	Workers for Iowa Department of Transportation (DOT)	*2 x 2 + 1 factorial design*			
		#1: control- no intervention			
		#2: no UV photo, ageing video			
		#3: no UV photo, skin cancer video			
		#4: UV photo, ageing video			
		#5: UV photo, skin cancer video			
2- and 12-month follow-up	100% male, 97% white, mean age 46.5 years				
Malak et al., 2011 [32]	n = 194	2 day training course on skin cancer prevention, identifying skin cancers and sun protection methods + reinforcement posters around village + distribution of wide-brimmed hats	No significant difference in proportion using sunglasses, hats, or long-sleeved shirts	No control group; retention rate not stated; self-reported data; culturally specific sample e.g. preference for scarfs prevents use of hats	IV
Pre-post test	Farmers living in a village in western Turkey		Significant increase in proportion of those using sunscreen (+9.5%; p = 0.001) and shade umbrella (+75.2%; p < 0.001); and decrease in proportion of those working in the sun at peak UV periods (−15.3%; p = 0.003)		
6-month follow up	44% male, 58% dark-skinned, mean age 39.1 years				
Woolley et al., 2008 [12]	n = 47	Case condition (n = 26): Employees in a workplace with long-standing mandatory sun protection policy	No significant differences in sun burns in past month; level of tanning on right hand or forearm, number of solar keratoses on right forearm, or usually wearing a wide-brim hat or sunscreen while at work	Limited power due to small sample size, did not adjust for potential covariates e.g. length of time spent working for organisation	III-2
Case control	Road workers and construction workers in Queensland, Australia	Control (n = 21): Employees in a workplace where sun protection is voluntary	Mandatory workplace employees had fewer solar keratoses on dorsum of right hand (0.3 vs 4.0, p = 0.006), less previously excised self-reported skin cancers (0.5 vs 3.5, p = 0.008), and were more likely to usually wear a long-sleeved shirt at work (81% vs 29%, p < 0.001)		
Single timepoint	89% male, mean age 42 years				
Anderson et al., 2008 [33]	n = 4,007 (39% retention)	Intervention: n = 13 ski areas received Go Sun Smart (GSS) Health Communication Campaign: advice/training to wear sun protection (sunscreen & protective lip-balm, hat, protective eyewear) delivered through workplace communication channels using 23 items including posters, magnets, website, newsletter articles, training programs for managers	At 6-month follow up, significantly less reported sunburn > =1 over past summer in intervention group (50%) compared to control (53%, p = 0.01)	Fluctuating study population due to nature of the organisation; low retention rate; implementation of program varied per ski area	III-2
Pair-matched group-randomised before and after controlled design	Ski area employees, in 26 ski areas in Western USA and Canada.	Control: n = 13 ski areas did not receive GSS	Significantly better sun protection scale *(combined average of sun protection behaviours: sunscreen; lip-balm; protective clothing; hat; sunglasses/goggles; limit time in sun; stay in shade; have sunscreen, sunglasses and hat with them at all times; watch skin closely to avoid sunburning)* in intervention group compared to control (3% adjusted difference, p = 0.04)		
3- and 6-month follow-up	64% male, 96% white, average age 34 years				
Mayer et al., 2007 [34]	n = 2,662 (82% retention)	Intervention: 35 postal stations (n = 1,257) received SUNWISE sun safety program: provision of wide-brim hats and sunscreen, sun safety educational sessions and visual cues prompting sun safe reminders	Significant increase in proportion who always use sunscreen at 2-years in intervention group (+12%) compared to control (+3%)		II
RCT	Letter-carriers at 70 US postal stations in 3 geographic regions in Southern California, USA	Control: 35 postal stations (n = 1,405) did not receive SUNWISE sun safety program.	Significant increase in proportion who always wear a wide-brim hat at 2-years in intervention group (+13%) compared to control (+1%)		
3-month, 1- and 2-year follow-up	70% male, mean age 43.0 years				
Andersen et al., 2012 [35]	n = 2228	Intervention (n = 33 ski areas): BDS (Basic Dissemination Strategy) + EDS (Enhanced Dissemination strategy) of Go Sun Smart (GSS)	No significant differences in sun protection scale or sunburn history between BDS and EDS groups	No pretest or adjustment for baseline levels of sun protection	III-2
Cluster-randomised post test only	Employees at 68 U.S and Canadian ski areas	Control (n = 35 ski areas): BDS only of GSS sun safety program.	Employees at organisations where 9+ of the 23 GSS items were used scored significantly higher on the sun protection scale compared to those where <4 GSS items were used (3% difference, p < 0.05)		
Disseminated over a single ski season in three waves (2004, 2005, 2006)	64% male, mean age 35.7 years, 93% white				

^a.^Based on Australian National Health and Medical Research Council (NHMRC) evidence hierarchy [36].

## Results

**Figure 1:**
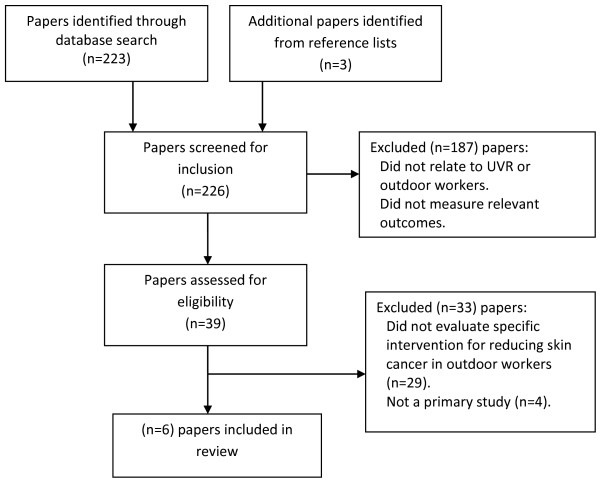
Summary of literature search and study selection.

Of 223 papers screened for inclusion, 38 were reviewed, and of these, six papers met criteria and were included in the review (Figure 1). Extracted data are displayed in Table 1.

### Populations studied

Studies represented interventions for workers from several outdoor industries: road workers [12,18], farmers [32], construction workers [12], ski area employees [33,35], and postal workers [34]. Most studies included predominantly male and fair-skinned white samples.

### Interventions used

Five of the six studies involved worksite based intervention strategies [12,18,33-35] while a single study measured a community based intervention [32]. One Australian study focused on workplace policy, comparing workers at a workplace with long-standing mandatory sun protection policy, to those at a workplace where sun protection was voluntary [12]. The remaining five interventions included an awareness raising or educational component, with varying information, delivery, and timeframes. A study of Turkish farmers used a one-off two day training course on sun protection, with reinforcement posters [32]. In another study using a one-off intervention, transport workers were given a UV photo of their skin and an educational video on either ageing or skin cancer risk [18]. Other studies used more intensive educational programs. The Go Sun Smart (GSS) communication strategy for snow ski area employees involved education and training on wearing sun protection delivered through workplace channels (23 items including posters, magnets, newsletter articles, website), with training programs for managers advocating sun safety practice to workers [35]. The SUNWISE program for postal service letter-carriers included six brief education sessions over a period of two years, about topics including skin cancer risk and sun protection strategies, accompanied by visual awareness cues such as magnets, key chains and posters [34]. In contrast to the other studies, the SUNWISE program involved a PPE component by providing broad-brimmed hats and sunscreen to workers [34]. Almost all interventions included an educational or awareness raising component, while differences included the delivery mode and intensity of that component, and the inclusion of structural or policy intervention components.

### Outcome measures and results

The main outcome measure was the proportion of participants increasing their self-reported and observed sun protection behaviours; or combined sun protection scores, which presented mixed results. A greater proportion of Turkish farmers used sunscreen (1.3% increased to 10.8%) and shade umbrellas (22.9% increased to 98.1%) 6 months following the intervention, although the proportion using hats, sunglasses and long-sleeved shirts did not increase (no control group included) [32]. In the study measuring workplace policy, a larger proportion of those in the mandatory policy organisation wore long-sleeved shirts (81% compared to 29%), but there was no difference in use of broad-brimmed hats or sunscreen [12]. Two years following the initiation of the SUNWISE program for postal workers, a larger increase in proportion of workers always wearing broad-brimmed hats and using sunscreen was observed in the intervention group (27%-40% for both broad-brimmed hats and sunscreen) [34]. In the control group the increase of wearing broad-brimmed hats (21-22%) and sunscreen (24%-26%) was noticeably less [34]. Of three studies measuring averaged sun protection scores, all found statistically significant increases in the intervention group compared to control at 6-12 month follow up [18,33,35].

Studies also used more objective measures of skin cancer risk reduction. The two studies measuring dissemination of the GSS program to ski area employees measured sunburn in the past season [33,35]. At 6-month follow-up in the initial trial, a lower proportion of workers in intervention areas reported being sunburnt one or more times over the past summer (50%) compared to those at ski areas which did not receive GSS (53%, p = 0.01) [33]. In the second trial, a similar proportion of workers at ski areas receiving GSS with a basic dissemination strategy had been sunburnt at least once over the past winter (26.0%) compared to workers at areas receiving GSS with an enhanced dissemination strategy (25.6%) [35]. In a workplace with mandatory sun protection policy, fewer solar keratoses on the dorsum of their right hand (0.3 vs 4.0), and fewer previously excised skin cancers (0.5 vs 3.5) were found, compared to workers in a voluntary sun protection workplace; however they had similar levels of tanning on right hand/forearm and number of solar keratoses on right forearm [12]. In the postal workers intervention, Mayer et al. used an objective measure of skin colour, finding the shade of tan decreased in both intervention and control groups over time [34]. There was no significant difference in this between the groups.

## Discussion

Outdoor workers are at risk of developing skin cancer and typically use inadequate levels of sun protection to mitigate this risk. In recent years, several large studies with extended follow-up times have demonstrated the efficacy of educational and multi-component interventions to increase sun protection. There is less evidence for the effectiveness of policy or specific intervention components.

Workplace policy documents provide guidelines for the day-to-day operation of an organisation, outlining expectations of both employers and employees [37]. Policies on sun protection often include guidelines on provision of PPE by employer, expectations of workers to use PPE, scheduling of tasks to avoid excessive exposure to UVR, use of shade, and/or education and training about sun protection [30]. Such policies may be enforced through disciplinary action (e.g. for not wearing PPE) or may be voluntary. There is currently limited evidence for the effectiveness of workplace sun protection policy alone in improving sun protection behaviour of workers. No known studies have initiated a sun protection policy as a single intervention; although one randomised trial used policy consultations alongside other intervention components with positive results [23]. One study included in the current review provided evidence that mandatory policy may increase some sun protective behaviours compared to a workplace where sun protection is voluntary [12]; but limitations to this study prevent any strong conclusions. In the same study, workers at the mandatory organisation used more sun protection while at work and reported high levels of sun exposure and low sun protection during their leisure time [12]. This study suggests enforcing sun protection at work does not necessarily have a flow on effect to sun protection in non-work time. It may have the opposite effect. Based on the theory of reactance [38], workers under pressure to conform to strict sun protection standards at work may respond by decreasing their protective behaviours when free to do so (i.e. in their leisure time). This highlights the importance of including workers in the development of policies to ascertain ownership of multi-component policies aimed at improving attitudes towards sun protection.

Three of the randomised trials reviewed provided evidence for the long-term efficacy of workplace-based interventions incorporating education and awareness about skin protection [18,33,34]. In a short appearance-based intervention, transportation workers exposed to a UV photograph and/or video information about skin cancer increased sun protection behaviour significantly, and this was maintained at 12 months [18]. While this study was targeted at individuals, two other studies (GSS for ski area employees, SUNWISE for postal workers) used organisation-wide dissemination strategies, also resulting in medium to long term improved sunscreen use and use of protective clothing [33,34]. Trials conducted previous to 2007 with shorter follow-up periods had mixed results: improvements in sun protection habits were observed following skin screening/education for Australian outdoor workers [28] and a training program for Hawaiian outdoor recreation staff [23]; Geller et al. [24] found no increase in sun protection following a training module for lifeguards, although significant reduction in sunburns was observed. Effectiveness may depend on how thoroughly the program is implemented; in the GSS trial, workers at ski areas where more of the 23 GSS items had been displayed reported more sun protection behaviours [33]. The short and long-term efficacy of education may depend on how appropriately programs are targeted and whether they are implemented alongside other components.

Based on ecological models of health behaviour [39], the provision of PPE and/or protective equipment (e.g. shade cloths) has been suggested to improve sun protection habits, by providing broader support to promote positive behaviours. PPE and environmental provisions are frequently recommended as a necessary component of sun protection policies [40,41]. In the two large-scale interventions mentioned above, the GSS for ski area employees used a communication strategy only, with no policy/structural changes or provision of PPE [33]. The SUNWISE program for postal workers provided PPE (hats and sunscreen) alongside education sessions [34]. Both trials had positive outcomes. The SUNWISE intervention demonstrated larger improvements using a less resource-intensive intervention. This may be due to the provision of PPE but the effects of individual intervention components were not measured in the study [34]. There is mixed evidence for the effectiveness of PPE/environmental considerations over education for improving sun protection behaviours. For example, in a study of outdoor recreation workers, Glanz et al. found both those who received an education-only program, and those receiving education and policy/environment program improved their sun protection behaviour, with no significant differences between groups [23]. The Hawthorne effect, which describes people changing their behaviour only as a result of feeling observed, may partly explain this similarity. By contrast, Azizi et al. [26] found the provision of PPE and education resulted in an increase in sun protection in Israeli workers. Both of these studies have limitations. Further research is necessary to demonstrate the need for PPE and protective equipment.

A further environmental consideration is the impact of workplace culture on sun protection behaviours. Cross-sectional studies suggest the importance of culture: two New Zealand studies [42,43] found workers who perceived sun protection was valued at their workplace had higher levels of sun protection. An American study reported a positive association between social norms and lifeguards’ sun protection [44]. However, there remains no specific prospective evidence for directly intervening on workplace culture to improve sun protection. Of the studies reviewed in this paper, a key component of both the GSS and SUNWISE programs was to encourage managers and opinion leaders in the workplace to model and recommend sun protection to workers. Neither study provided information on the outcome of this specific component [33,34].

In light of the growing evidence for the effectiveness of multi-component interventions, successful findings must be translated to practice to improve skin protection and decrease skin cancer in this high risk group. A single study included in this review compared two strategies for disseminating the GSS program for ski area employees to all ski areas across the USA [35]. As no pre-test was available in this study, actual effects of the dissemination cannot be measured. However, the results of the GSS efficacy study could indicate what one might expect from the programs influence [35]. In that study, at ski areas where more GSS items were displayed, workers reported higher sun protection [35]. Future research could take translational approaches such as those described in this study or other approaches. Health promotion researchers are increasingly using a participatory action research approach [45], which gives participants an active role in the research process, while implementing the intervention and measuring its effects [46].

## Conclusion

In recent years, large scale, quality research projects have provided further evidence for effective strategies to decrease skin cancer risk in outdoor workers; however, there is still work to be done. For example, the reviewed studies used different outcome measures of sun protection or skin cancer risk, making comparison difficult. Few studies measured the effect of individual intervention components, making it more challenging to discern the essential elements that need to be included in the future to design successful interventions. The reviewed studies identified educational and multi-component interventions more successful in increasing sun protection, with less evidence provided for the effectiveness of policy or specific intervention components. Finally, the largest studies focused on specific industries (postal service and ski areas) where worker characteristics and culture may differ from workers in other outdoor industries such as construction and farming. For example, the mean age of ski area employees was much lower than the mean age of construction employees in recent studies [12,17]). Studies including a diverse range of workers across different industries along with use of consistent gold standard assessment instruments are necessary. Further research is therefore required to ascertain the appropriateness of interventions in these workers.

## Abbreviations

BCC: Basal cell carcinoma; SCC: Squamous cell carcinoma; NMSC: Non-melanoma skin cancer; UVR: Ultraviolet radiation; GSS: Go Sun Smart program; PPE: Personal Protective Equipment; RCT: Randomised controlled trial; BDS: Basic dissemination strategy; EDS: Enhanced dissemination strategy.

## Competing interests

The authors declare that they have no competing interests.

## Authors’ contributions

MJ and JA coordinated and initiated the manuscript. JA and CH screened papers for eligibility for inclusion. MJ, JA and CH participated in the design of the study and conducted the literature review. All authors provided critical review and final approval.
